# A randomized controlled trial testing the efficacy of a Nurse Home Visiting Program for Pregnant Adolescents

**DOI:** 10.1038/s41598-021-93938-7

**Published:** 2021-07-13

**Authors:** Daniel Fatori, Pedro Fonseca Zuccolo, Elizabeth Shephard, Helena Brentani, Alicia Matijasevich, Alexandre Archanjo Ferraro, Lislaine Aparecida Fracolli, Anna Maria Chiesa, James Leckman, Euripedes Constantino Miguel, Guilherme V. Polanczyk

**Affiliations:** 1 Departamento de Psiquiatria, Faculdade de Medicina FMUSP, Universidade de São Paulo, R Dr Ovídio Pires de Campos, 785, São Paulo, SP CEP 05403-903 Brazil; 2 Departamento de Medicina Preventiva, Faculdade de Medicina FMUSP, Universidade de São Paulo, São Paulo, SP Brazil; 3 Departamento de Pediatria, Faculdade de Medicina FMUSP, Universidade de São Paulo, São Paulo, SP Brazil; 4 Departamento de Enfermagem Em Saúde Coletiva da Escola de Enfermagem, Universidade de São Paulo, São Paulo, SP Brazil; 5 Yale Child Study Center, Yale University School of Medicine, New Haven, CT USA

**Keywords:** Paediatric research, Randomized controlled trials

## Abstract

To test the efficacy of a nurse home visiting program (HVP) on child development, maternal and environmental outcomes in the first years of life. We conducted a randomized controlled trial to test the efficacy of *Primeiros Laços*, a nurse HVP for adolescent mothers living in a poor urban area of São Paulo, Brazil. Eighty adolescent mothers were included and randomized to receive either *Primeiros Laços* (intervention group, n = 40) or healthcare as usual (control group, n = 40). *Primeiros Laços* is a home visiting intervention delivered by trained nurses that starts during the first 16 weeks of pregnancy and continues to the child’s age of 24 months. Participants were assessed by blind interviewers at 8–16 weeks of pregnancy (baseline), 30 weeks of pregnancy, and 3, 6, 12, and 24 months of child’s age. We assessed oscillatory power in the mid-range alpha frequency via electroencephalography when the children were aged 6 months. Child development was measured by the Bayley Scales of Infant Development Third Edition (BSID-III). Weight and length were measured by trained professionals and anthropometric indexes were calculated. The home environment and maternal interaction with the child was measured by the Home Observation and Measurement of the Environment. Generalized estimating equation models were used to examine intervention effects on the trajectories of outcomes. Standardized effect sizes (Cohen’s d) were calculated using marginal means from endpoint assessments of all outcomes. The trial was registered at clinicaltrial.gov: NCT02807818. Our analyses showed significant positive effects of the intervention on child expressive language development (coefficient = 0.89, 95% CI [0.18, 1.61], *p* = 0.014), maternal emotional/verbal responsivity (coefficient = 0.97, 95% CI [0.37, 1.58], *p* = 0.002), and opportunities for variety in daily stimulation (coefficient = 0.37, 95% CI [0.09, 0.66], *p* = 0.009). Standardized effect sizes of the intervention were small to moderate. *Primeiros Laços* is a promising intervention to promote child development and to improve the home environment of low-income adolescent mothers. However, considering the limitations of our study, future studies should be conducted to assess *Primeiros Laços* potential to benefit this population.

**Clinical Trial Registration**: The study was registered at clinicaltrial.gov (Registration date: 21/06/2016 and Registration number: NCT02807818).

## Introduction

The first years of life are fundamental for the cognitive, social and emotional development of the child, deeply impacting later life outcomes^1,2^. Educational outcomes, such as dropout, repetition and school performance, reading, and arithmetic skills, are associated with cognitive development in early childhood^3^. Adults that early in life received adequate stimulation and high quality education have better physical and mental health, higher income, more years of schooling, and a lower rate of involvement in crime than individuals who did not receive these types of interventions^4–6^.

Child development is influenced by psychosocial, biological and genetic factors. In this period, specifically, the brain is at a critical stage, developing rapidly and dynamically^7^. During this process, the environment, alongside genetics, plays a crucial role, which may facilitate or impair brain development^8,9^. In this context, poverty is known to be a decisive factor in determining the quality of the environment, as well as the presence of many risk factors. Children of poor families have less access to resources such as books and computers, often live in overcrowded houses and neighborhoods with high levels of criminality, and have greater exposure to intrafamilial violence^10^. Also, they are more likely to suffer intrauterine growth restriction, developmental problems, being born preterm and with low weight^11,12^. A low family socioeconomic level during childhood is associated with various negative health outcomes in adult life, such as cardiovascular disease, obesity, alcohol/substance abuse, and mortality related to cancer, diabetes, respiratory, and cardiovascular diseases^13,14^.

Poverty is frequently associated with adolescent pregnancy, another important risk factor for various maternal and child negative outcomes^15,16^. Children of adolescent mothers often have impaired development in domains such as cognition, language, schooling, socioemotional and behavioral functioning^17–20^. Adolescent mothers, later in life, report lower educational level and income^21^. There is a higher prevalence of depression in adolescent mothers when compared to adult mothers^22^, a condition associated with negative effects on quality of child stimulation^23^. Regarding mother–child interaction, adolescent mothers are less sensitive to the child’s needs, provide less verbal stimulation, and have higher rates of insecure and disorganized attachment^17,24^. They also show more parental stress when compared to adult mothers^25^. As a result, adolescent pregnancy is a major component of the cycle of health problems and poverty, especially in developing countries, where an intergenerational impact has been described^26^. To break this cycle, evidence-based interventions that aim to address the specific needs and challenges of adolescent mothers and their children are needed^27^.

Thus, it is paramount to develop and test interventions focused on adolescent mothers living under adverse conditions to ensure positive maternal and child outcomes. To maximize its effects, it is key that early childhood development interventions start in the prenatal period^28^. During gestation, factors such as substance use and nutritional deficiency can negatively affect the child's brain development and even cause malformations^29,30^. A number of studies have shown that prenatal care can reduce infant mortality and the occurrence of low birth weight^28,31^.

From this perspective, intervention programs involving frequent and continuous home visits by health professionals from gestation to the first years of the child’s life have shown for decades to effectively promote maternal health and child development. Home visiting programs (HVP) are aimed at modifying risk factors for multiple maternal and child adverse outcomes. There is a wide range of modifiable risk factors to intervene during pregnancy, such as substance use and nutritional deficiency since both can negatively affect child brain development^29,30^. Also, cognitive stimulation and adequate parenting skills during the first years of life are important to prevent child development and behavior problems^32,33^. HVP can have positive long-lasting effects on income, schooling, mental health problems, and antisocial behavior, among others^34,35^.

A well-known example of an evidence-based HVP is the Nurse-Family Partnership (NFP)^36^. The NFP is a pioneering program of prenatal and postnatal home visits (up to two years of age) by nurses for impoverished women with the following objectives: promoting healthy maternal behaviors, improving health and development of children through appropriate parental care, and improving the mother's life course development by encouraging family, professional, and life planning. The group of mothers who received the intervention, when compared to the usual treatment group, had fewer subsequent pregnancies and longer interpregnancy intervals, longer relationship with the partner, less social services use, children with better developmental outcomes and fewer mental health problems^37^, less child abuse and neglect^38^, less intimate partner violence^39^, and reduced child mortality^40^.

However, evidence-based HVP for adolescent mothers are scarce in low- and middle-income countries (LMIC), where the majority of adolescent births occur^41,42^. It is estimated that adolescent birth rates are four times higher in the poorest regions of the world when compared to high-income regions^43^. Furthermore, 35.8% of 3 to 4-year-old children fail to reach basic cognitive and socioemotional milestones in LMICs^44^. This context led us to develop *Primeiros Laços*, a nurse HVP for adolescent mothers living in a deprived urban area of São Paulo, Brazil, aimed at promoting child development and fostering mother–child relationships.

We hypothesized that children of adolescent mothers that received the *Primeiros Laços* program, when compared to children of adolescent mothers who did not receive the intervention, would show significant improvements in child development markers in the first 2 years of life: (1) oscillatory alpha power, an electroencephalography (EEG) measure of brain development, (2) cognitive, language, and motor developmental milestones measured by the Bayley Scales of Infant Development Third Edition (BSID-III), and (3) child growth measured by anthropometric indices. We also hypothesized our intervention would positively impact (4) the quality of stimulation and support available to the child in the home environment measured by the Home Observation for Measurement of the Environment (HOME). Therefore, the objective of the present study is to test the efficacy of *Primeiros Laços* on child development, maternal and environmental outcomes in the first 2 years of life.

## Methods

### Design

We conducted a parallel-group randomized controlled trial to test the efficacy of a nurse HVP for adolescent mothers living in a poor urban area in Sao Paulo, Brazil.

### Participants

From June to September 2015, eighty low-income pregnant youth were randomized into two groups: intervention (HVP by nurses, n = 40) and control (healthcare as usual, n = 40). Inclusion criteria were: (a) first pregnancy, (b) pregnant youth aged 14–19 years old, (c) low socioeconomic status (classes C, D, E according to the ABEP scale)^45^, (d) pregnancy between 8 and 16 weeks, (e) living in the western region of Sao Paulo. Randomization was stratified by the following characteristics: primary healthcare unit type, and grandmother years of schooling. The allocation ratio used was 1:1. An on-site research manager hired specifically for this study was responsible for randomizing and allocating participants.

### Setting

The western region of Sao Paulo city is characterized by high rates of violence, widespread slums, and adverse living conditions. In 2015, the population of the western region was 395 thousand inhabitants with approximately 20.0% of adolescents. Child mortality rate was 5.7–12.4 per 1000 live births and neonatal mortality was 1.7–2.9 per 1000 live births, depending on the district. At the time, the homicide rate ranged from 3.7 to 8.6 per 100 thousand inhabitants^46^. We established a facility within the community at Liga Solidaria’s Educandário Dom Duarte (ligasolidaria.org.br), a well-known institution with decades of experience in educational and social services, where our research staff coordinated the study and conducted blinded assessments.

### Procedures

Participants were identified in Primary Healthcare Units in the western region of Sao Paulo. Every potential participant received a brochure with a brief explanation of the study inviting them for an interview with a research assistant. All participants who met inclusion criteria and were interested in the study signed a consent form approved by the institutional review boards of the University of Sao Paulo Medical School (ref: 052/15) and the São Paulo Municipal Health Department.

Participants were assessed by blinded interviewers at 8–16 weeks of pregnancy (baseline), 30 weeks of pregnancy, and 3, 6, 12, and 24 months of infant’s age. All interviewers were experienced psychologists who underwent a one-month training provided by study coordinators. Prior to the baseline assessment, interviewers pilot-tested the protocol assessing voluntary participants living in the Western region.

### Sample characterization measures

Sample characterization at baseline consisted of information on maternal age, ethnicity, school enrollment, educational level, occupation, grandmother’s education level, family enrollment in social welfare program, number of people living in the residence, and total family income per month.

Maternal mental health was measured using the Beck Depression Inventory (BDI)^47,48^, the Beck Anxiety Scale (BAI)^49^, and the Adult Attention Deficit Hyperactivity Disorder (ADHD) Self-report Scale (ASRS)^50^. Those are well-known scales validated in Brazil showing adequate psychometric properties^51–53^. To characterize the sample at baseline, we used the following cut-offs for each domain/scale: depression (BDI score > 20), anxiety (BAI score > 16), and ADHD (ASRS score > 9). Also, substance, alcohol, and tobacco use (lifetime and during gestation) was measured by a structured questionnaire developed for this study.

The Childhood Trauma Questionnaire (CTQ)^54^ was administered to participants to collect information on maternal history of abuse. The CTQ screens for experiences of abuse and neglect during childhood, resulting in five domains: emotional, physical, sexual, emotional neglect, and physical neglect. It has been translated and validated in Brazil^55,56^.

Family food insecurity was measured by the brief version of the Brazilian Food Insecurity Scale (Escala de Insegurança Alimentar, EBIA)^57^, that is based on a widely used scale in Brazil developed to measure food insecurity at the national level with the objective of providing reliable data for federal programs aimed at eliminating hunger^58^.

### Intervention

*Primeiros Laços* is a home visiting intervention delivered by trained nurses based on three theoretical frameworks: attachment theory^59^, self-efficacy theory^60^, and the bioecological model^61^. *Primeiros Laços* was based on the Brazilian program *Janelas de Oportunidades*^62,63^, the Minding the Baby program^64^, and the NFP^36^, with input from key national stakeholders involved in early childhood and maternal health research and advocacy. The program targets first-time pregnant adolescents and their child, starting during the first 16 weeks of pregnancy and continuing until the child’s age of 24 months. It has a learning-based approach aimed at helping the mother prioritize the child in her life in order to improve maternal sensitivity to the child’s behaviors and emotions. Nurses visited participants according to the following plan: (a) biweekly during gestation and from 2 to 20 months of child’s age, (b) weekly during the first and last month of pregnancy, and the puerperium, and (c) monthly from 21 to 24 months of child’s age.

The *Primeiros Laços* content is directed to five domains: (1) health and social care: promotion of maternal and child health focused on nutrition, hygiene, common pathologies in childhood, domestic care, vaccination, prevention of accidents, and early child development; (2) environmental health: support to ensure mothers have adequate living conditions, safe housing, access to health services, and daycare and school for children; (3) life course: support to help adolescent mothers plan future goals, such as finishing high school, finding a part-time job, finding a career, planning to go to college, and postpone the birth of a second child. Life-course planning was discussed individually according to personal goals and objectives; (4) parenting skills: education on child development and parenting skills with the objective of helping the adolescent mother to develop healthy mother–child interactions in order to ensure a secure attachment style; (5) family and social support: the role of family members and friends to support the needs of mother–child dyad was highlighted. Family members living with the adolescent mother or in the community were also invited to home visits to be part of the intervention process. Nurses were supervised weekly by senior nurses (A.C., L.F.) and a child psychologist. More details about the intervention can be found elsewhere^64–67^.

### Care as usual

Participants allocated to the control group received healthcare from the Unified Health System (Sistema Único de Saúde, SUS), Brazil’s public health system^68^, according to national guidelines^68–71^ that are in line with the World Health Organization guidelines. The primary care system delivers prenatal and postnatal care with an emphasis on the prevention of gestational problems. Cases of gestational risk were referred to specialized health services.

### Outcome measures

We assessed oscillatory power in the infant alpha frequency (6–9 Hz) during a quasi-resting-state condition (while infants watched videos of abstract shapes) using EEG when the children were aged 6 months. The data were recorded using a 128-channel Geodesic Sensor Net and a NetAmp 200 DC-coupled amplifier (Electrical Geodesics Inc., Eugene, OR, USA). Absolute power in the alpha band (6–9 Hz) was computed for each electrode and subsequently averaged across clusters of electrodes in the frontal, central, right-hemisphere posterior, left-hemisphere posterior and occipital scalp regions, following previous work^72^. Alpha power in each cluster was used in analysis. EEG data collection was attempted with 50 children at age 6 months. Of these, 19 were excluded due to technical problems with the EEG system that resulted in corrupted data files (n = 4), poor-quality recordings (n = 10) or fewer than 20 artefact-free epochs (n = 5). Final analyses were therefore conducted on 31 participants (17 intervention, 14 control). The groups did not differ in the number of artefact-free epochs included in analysis (intervention mean = 48.41, SD = 19.29; control mean = 44.86, SD = 19.01; t(29) = − 0.51, *p* = 0.610). For a complete description of EEG methods, see the online Supplementary Methods.

Child development was assessed with the third edition of the Bayley Scales of Infant Development Third Edition (BSID-III)^73^ administered by a trained psychologist at 3, 6, 12, and 24 months of the child’s age. The BSID-III consists of a series of play tasks and behavior observations and usually takes between 45 and 60 min to administer. The BSID-III has been translated to Brazilian Portuguese and validated^73–76^, showing good psychometric properties. We assessed motor, language, and cognitive development. Since there is no Brazilian normative data available for the BSID-III raw scores were used in all analyses.

Child anthropometric growth was measured at birth, 6, 12, and 24 months of the child’s age. We measured child weight and length/height using standard procedures. Children’s BMI-for-age and length-for-age were calculated based on WHO growth standards^77^ via WHO Anthro version 3.2.2 SPSS syntax^78^. Z-scores ≤ 4 and ≥ 4 were excluded because those were considered measurement errors (BMI-for-age of 1 participant time-point from intervention, length-for-age of 1 participant time-point from control).

The quality of stimulation and support available to the child in the home environment was assessed via the Home Observation for Measurement of the Environment (HOME)^79,80^. HOME is a 45-item instrument aimed at assessing the caring environment where the child is being reared. Information is obtained via observation of the home environment and interview questions with the primary caregiver. The scores can be divided into six domains: emotional and verbal responsivity, avoidance of restriction and punishment, organization of the physical/temporal environment, provision of appropriate play materials, parental involvement with the child, and opportunities for variety in daily stimulation. It was translated to Brazilian Portuguese and it has been applied in different regions and contexts in the country^80–83^.

### Sample size

The sample size was calculated based on the difference in EEG alpha frequency power between the groups (30%) with a probability of type I error of 5% and statistical power of 80%.

### Statistical analysis

EEG data were analysed using one ANOVA model with the between-subjects factor group (intervention vs. control) and the within-subjects factor electrode cluster (frontal, central, right-hemisphere posterior, left-hemisphere posterior, occipital) with alpha power as the dependent variable. A significant interaction between group and electrode cluster was further investigated with Bonferroni-corrected planned pairwise contrasts between the groups at each electrode cluster.

Generalized estimating equation (GEE)^84^ models were used to examine intervention effects on the outcomes of interest that were measured longitudinally (child development and anthropometric growth, and quality of stimulation and support available to the child in the home environment). We used quasi-likelihood under the independence model criterion (QIC) to detect covariance structures that were optimal for unequally spaced observations for each model. Time was entered as a continuous covariate in all models. Fitted models were used to estimate and plot marginal mean scores for continuous outcomes at each time point. Time trends were verified by checking if a quadratic term was statistically significant^85,86^. We used a modified intention-to-treat approach in our analysis: all randomized participants were included in the analyses, except participants with missing data at all time points. Statistical tests were 2-sided and *p* values < 0.05 were considered statistically significant. Parameters were reported with 95% confidence intervals (CI). Standardized effect sizes (Cohen’s d) were calculated using marginal means from endpoint assessments of all outcomes, except the alpha power analysis, where we reported the partial η^2^. Cohen’s d were interpreted using the following reference values: 0.2, 0.5, 0.8 for small, medium, and large, respectively^87^. Partial η^2^ was interpreted using the following reference values: 0.01, 0.06, 0.14 for small, medium, and large, respectively^88^.

### Ethical aspects

The present study was approved by the Ethics Committee of the University of São Paulo Medical School (ref: 052/15), the University Hospital of the University of São Paulo, and by the Sao Paulo Municipal Health Department. Signed informed consent was given to participants and their primary caregivers. All methods were performed in accordance with the relevant guidelines and regulations. The study was registered at clinicaltrial.gov (Registration date: 21/06/2016 and Registration number: NCT02807818). No relevant changes to methods, design or outcomes after trial commencement were made. The full study protocol can be sent upon request.

## Results

### Recruitment and retention

**Figure 1:**
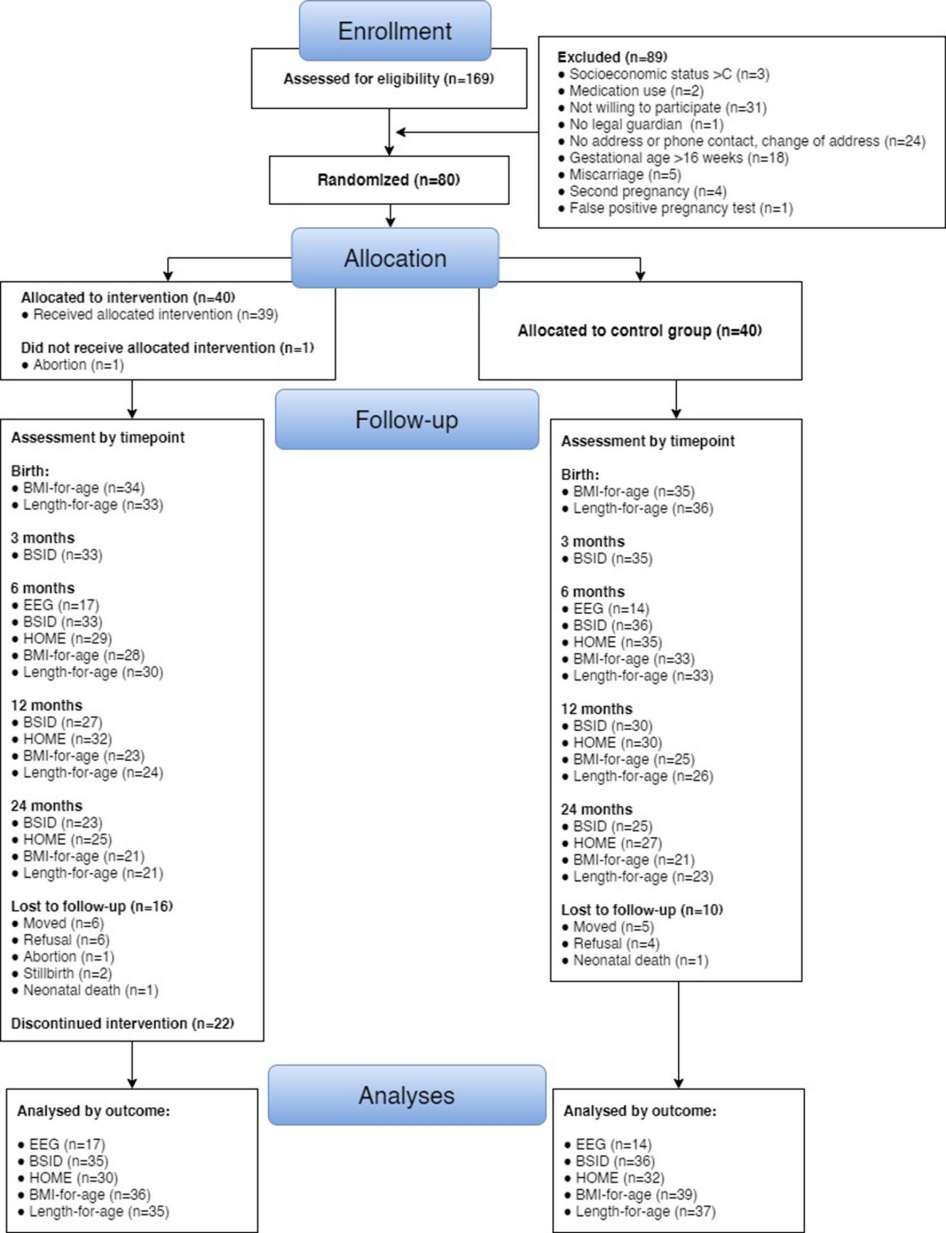
CONSORT flow diagram of the randomized controlled trial.

From June 2015 to March 2016, one hundred and sixty-nine pregnant youth were assessed for eligibility criteria in a face-to-face interview and 80 youth were included in the study. Participants were randomized to intervention (n = 40) and control (usual care, n = 40) groups. See CONSORT diagram below (Fig. 1). Intervention dropout rate was 40% (n = 16) at 24-months of child’s age. Mean number of nurse home visits in the intervention group was 38.3 (SD 21.3) at 24 months. A total of 66 nurse home visits were planned to be delivered by the time the children were 24-months old. 67.5% (n = 27) received at least 50% of planned nurse home visits.

### Sample characteristics

Table 1 describes participant and family sample characteristics at baseline. Maternal mean age was 17.1 (SD 1.2). 43.8% (n = 35) were enrolled in school. Twenty-one participants (26.3%) were enrolled in a government welfare program (i.e., *Bolsa Família*), 15 participants (18.8%) had a family income between 0 and 800 reais (equivalent to 0–1.645 in purchasing power parities^89^), 34 participants (42.5%) had food insecurity problems at home. Maternal mental health characteristics had the following distribution: 21.3% of depression, 23.8% of anxiety, 13.8% of ADHD. Participants from intervention and control groups did not differ statistically on any baseline characteristic.

**Table 1:** Baseline characteristics of the sample.

Characteristics	Control (N = 40)	Intervention (N = 40)	Total (N = 80)	*p* value
Maternal age	17.3 (1.2)	16.9 (1.3)	17.1 (1.2)	0.248
Maternal ethnicity, White	13 (32.5%)	12 (30.0%)	25 (31.3%)	1.000
Mother is enrolled in school	19 (47.5%)	16 (40.0%)	35 (43.8%)	0.652
Grandmother educational level, Illiterate/Inc. primary education	21 (52.5%)	16 (40.0%)	37 (46.3%)	0.370
Maternal educational level, Illiterate/Inc. primary education	4 (10.0%)	8 (20.0%)	12 (15.0%)	0.348
Maternal occupation, Working for pay	10 (25.0%)	5 (12.5%)	15 (18.8%)	0.252
Family enrolled in social welfare program	11 (27.5%)	10 (25.0%)	21 (26.3%)	1.000
Number of people living in the residence	3.6 (1.4)	3.6 (1.9)	3.6 (1.7)	0.947
Family income, 0–800 reais	7 (18.9%)	8 (22.2%)	15 (18.8%)	0.778
Maternal lifetime police problems	3 (7.5%)	5 (12.8%)	8 (10.1%)	0.481
Presence of family food insecurity	13 (32.5%)	21 (52.5%)	34 (42.5%)	0.113
Maternal history of substance use	12 (30.0%)	19 (47.5%)	31 (38.8%)	0.168
Presence of maternal chronic disease	3 (7.5%)	5 (12.5%)	8 (10.0%)	0.712
Presence of maternal sexually transmitted disease	2 (5.0%)	6 (15.0%)	8 (10.0%)	0.263
Maternal Self-efficacy	29.1 (6.1)	30.0 (7.4)	29.6 (6.8)	0.589
Maternal Depression^a^	7 (17.5%)	10 (25.0%)	17 (21.3%)	0.586
Maternal Suicidal Ideation	1 (2.5%)	2 (5.0%)	3 (3.8%)	1.000
Maternal Anxiety^b^	7 (17.5%)	12 (30.0%)	19 (23.8%)	0.311
Maternal ADHD^c^	4 (10.0%)	7 (17.5%)	11 (13.8%)	0.518
Maternal history of emotional abuse^d^	6 (15.0%)	8 (20.0%)	14 (17.5%)	0.770
Maternal history of physical abuse^e^	3 (7.5%)	4 (10.0%)	7 (8.8%)	1.000
Maternal history of sexual abuse^f^	3 (7.5%)	2 (5.0%)	5 (6.3%)	1.000
Maternal history of emotional neglect^g^	7 (17.5%)	2 (5.0%)	9 (11.3%)	0.729
Maternal history of physical neglect^h^	3 (7.5%)	3 (7.5%)	6 (7.5%)	1.000

Data depicted as mean (SD) or N (%).

*ADHD* attention-deficit/hyperactivity disorder, *ASRS* ADHD Self-report scale, *BAI* Beck Anxiety Inventory, *BDI* Beck Depression Inventory, *CTQ *Childhood trauma questionnaire, *Inc* incomplete, *M* mean, *SD* standard deviation.

^a^BDI moderate or severe levels (score ≥ 20); ^b^BAI moderate or severe levels (score ≥ 16); ^c^ASRS clinical level (score ≥ 9); ^d^CTQ moderate or severe levels (score > 13); ^e^CTQ moderate or severe levels (score ≥ 10); ^f^CTQ moderate or severe levels (score ≥ 8); ^g^CTQ moderate or severe levels (score ≥ 15); ^h^CTQ moderate or severe levels (score ≥ 10).

### Intervention effects

**Figure 2:**
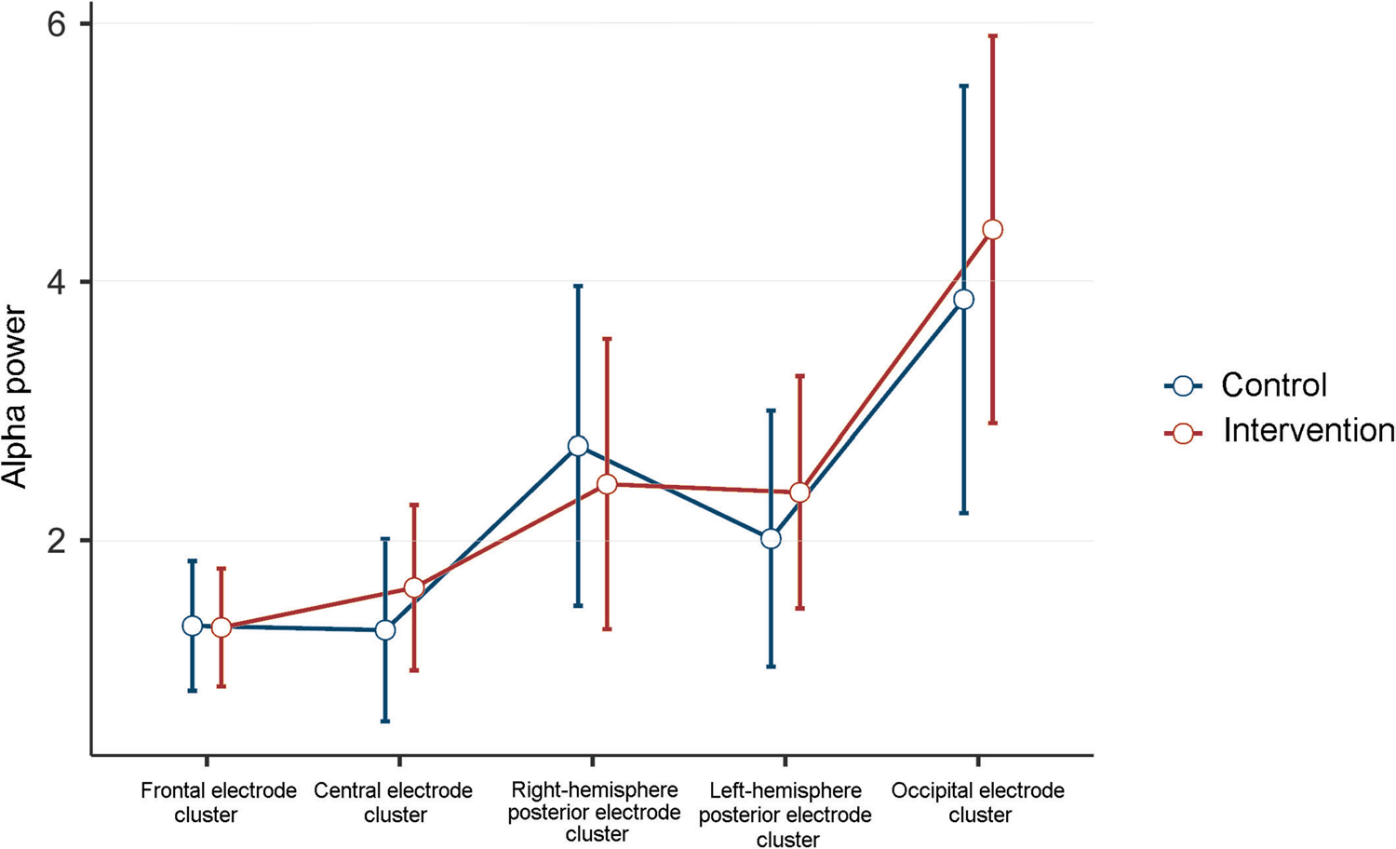
Intervention effects on child alpha power at age 6 months measured by EEG (intervention n = 17, control n = 14). Fitted model plot over time by electrode cluster and randomization status (*p* = 0.633, partial η^2^ = 0.02).

Oscillatory power in the mid-range alpha frequency measured with EEG was assessed at age 6 months (intervention n = 17, control n = 14). The groups did not differ significantly in alpha power at age 6 months (main effect of group: F(1,29) = 0.09, *p* = 0.768, partial η^2^ = 0.003; group × electrode cluster interaction: F(4,116) = 0.642, *p* = 0.633, partial η^2^ = 0.02). Mean alpha power values are presented by electrode cluster and group in Fig. 2.

**Figure 3:**
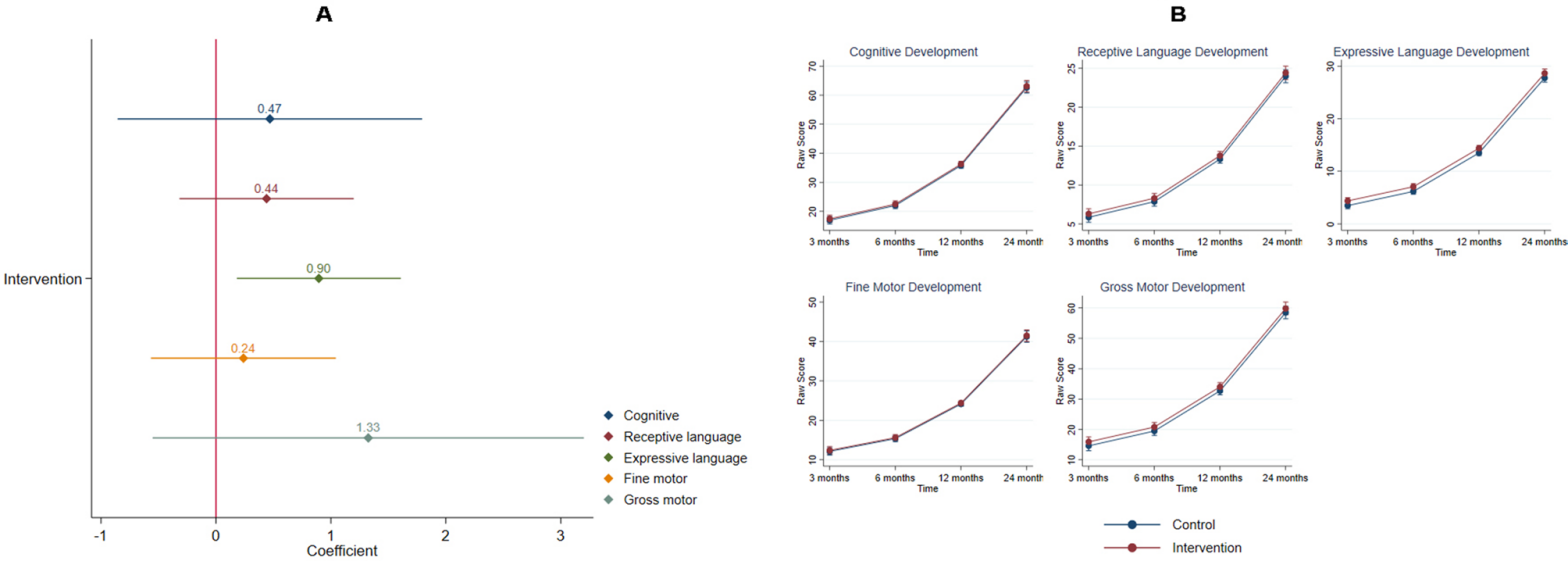
Intervention effects on child development measured by the BSID-III (Intervention 35, control 36). (**A**) Generalized estimating equations models results had the following parameters for each child development domain: cognitive development (coefficient = 0.46, 95% CI [0.85, 1.79], *p* = 0.488), receptive language development (coefficient = 0.44, 95% CI [− 0.32, 1.19], *p* = 0.255), expressive language development (coefficient = 0.89, 95% CI [0.18, 1.61], *p* = 0.014), fine motor development (coefficient = 0.23, 95% CI [− 0.56, 1.04], *p* = 0.561), gross motor development (coefficient = 1.32, 95% CI [− 0.55, 3.20], *p* = 0.166). (**B**) Fitted models plots of all child development domains over time by randomization status. Standardized effect sizes (Cohen’s d) were calculated from endpoint marginal means: cognitive development (d = 0.08), receptive language development (d = 0.17), expressive language development (d = 0.37), fine motor development (d = 0.05), gross motor development (d = 0.21).

Child development was assessed longitudinally at 3, 6, 12, and 24 months of child’s age (intervention n = 35, control n = 36). Figure 3 depicts intervention effects on child development measured by the BSID-III analyzed by GEE models. Expressive language development was significantly associated with the intervention (coefficient = 0.89, 95% CI [0.18, 1.61], *p* = 0.014). Estimated marginal means of expressive language scores of the intervention group were higher than the control group at all time points. Standardized effect size (d = 0.37) showed the intervention had a small to moderate effect on expressive language development.

**Figure 4:**
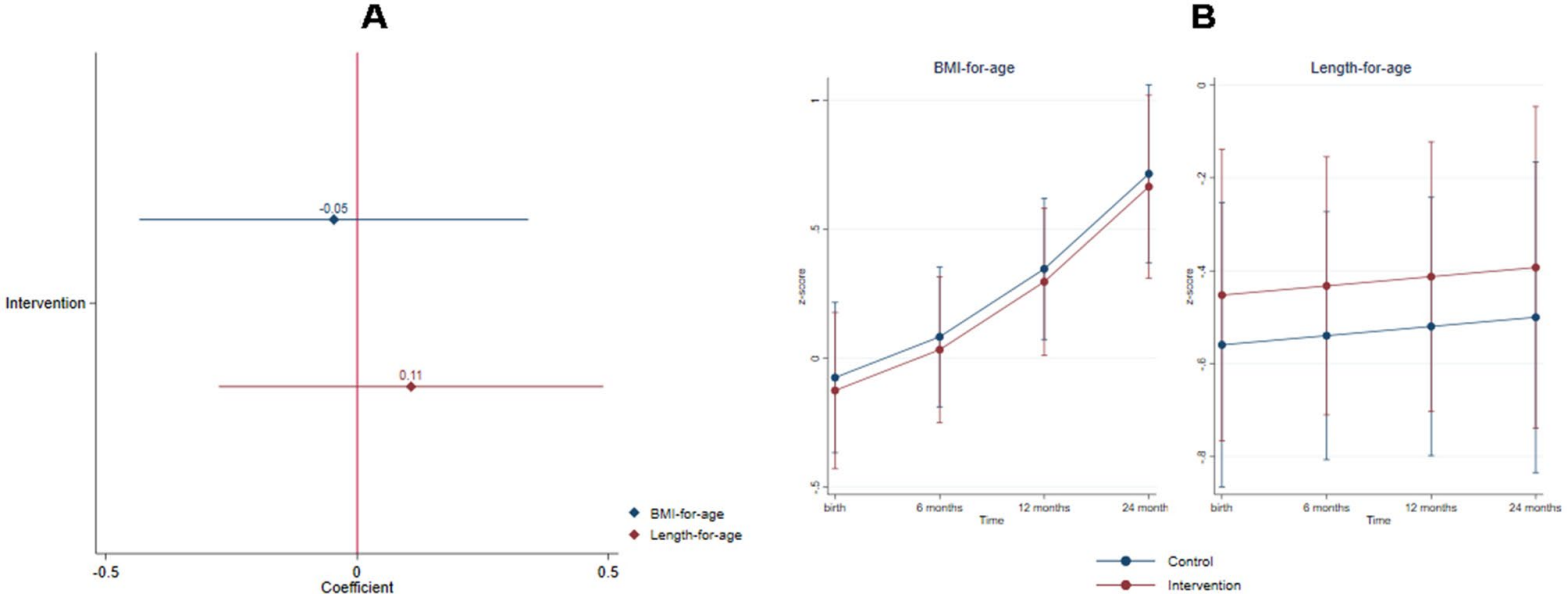
Intervention effects on anthropometric development (BMI-for-age: intervention n = 36, control n = 39; Length-for-age: intervention n = 35, control n = 37). (**A**) Generalized estimating equations models results had the following parameters for each child anthropometric development measure: BMI-for-age (coefficient = − 0.05, 95% CI [− 0.44, 0.34], *p* = 0.800), length-for-age (coefficient = 0.10, 95% CI [− 0.27, 0.49], *p* = 0.583). (**B**) Fitted models plots of all child development domains over time by randomization status. Standardized effect sizes (Cohen’s d) were calculated from endpoint marginal means: BMI-for-age (d = − 0.05), length-for-age (d = 0.10).

Intervention effects on child anthropometric development are depicted in Fig. 4 (BMI-for-age: intervention n = 36, control n = 39; Length-for-age: intervention n = 35, control n = 37). GEE models did not show statistically significant effects of the intervention on BMI-for-age and length-for-age (Fig. 3). Table S3 depicts length-for-age and BMI-for-age categorized by z-score. At 24 months of age, the majority of the sample was in the normal range of length-for-age (86.4%) and BMI-for-age (57.1%).

**Figure 5:**
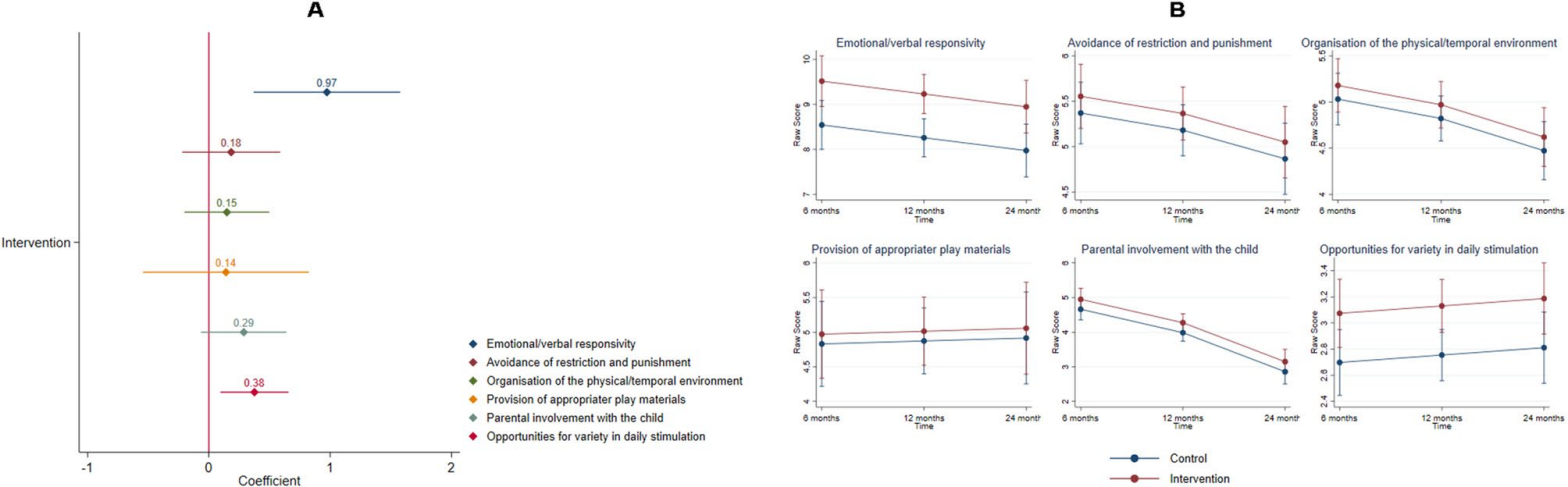
Intervention effects on mother–child relationship and home environment measured by the HOME (intervention n = 30, control n = 32). (**A**) Generalized estimating equations models results had the following parameters for each HOME domain: emotional/verbal responsivity (coefficient = 0.97, 95% CI [0.37, 1.58], *p* = 0.002), avoidance of restriction and punishment (coefficient = 0.18, 95% CI [− 0.22, 0.59], *p* = 0.372), organization of the physical/temporal environment (coefficient = 0.14, 95% CI [− 0.20, 0.49], *p* = 0.406), provision of appropriate play materials (coefficient = 0.14, 95% CI [– 0.54, 0.82], *p* = 0.687), parental involvement with the child (coefficient = 0.28, 95% CI [– 0.06, 0.64], *p* = 0.110), opportunities for variety in daily stimulation (coefficient = 0.37, 95% CI [0.09, 0.66], *p* = 0.009). (**B**) Fitted models plots of all HOME domains over time by randomization status. Standardized effect sizes (Cohen’s d) were calculated from endpoint marginal means: emotional/verbal responsivity (d = 0.55), avoidance of restriction and punishment (d = 0.16), organization of the physical/temporal environment (d = 0.16), provision of appropriate play materials (d = 0.07), parental involvement with the child (d = 0.27), opportunities for variety in daily stimulation (d = 0.46).

Figure 5 shows the intervention effects on the mother–child relationship and the home environment measured by the HOME scale (intervention n = 30, control n = 32). Maternal emotional and verbal responsivity (coefficient = 0.97, 95% CI [0.37, 1.58], *p* = 0.002) and opportunities for variety in daily stimulation (coefficient = 0.37, 95% CI [0.09, 0.66], *p* = 0.009) were significantly associated with the intervention, both showing standardized effect sizes in the medium range (d = 0.55 and d = 0.46, respectively).

Table S1 reports the number of participants included in the analyses for each outcome. Only participants without data in at least one time point were not included in the analyses (e.g., stillbirth, neonatal death, etc.). Differences at baseline between participants included vs. not included in the efficacy analyses were tested (Table S2). The majority of participants enrolled in school at the time of the baseline assessment provided information on outcomes of the study. Maternal age (*p* = 0.004), mother enrolled in school (*p* = 0.005), and maternal history of physical abuse (*p* = 0.012) were associated with inclusion in the EEG outcome analysis. Mother enrolled in school (p = 0.004) and maternal history of emotional abuse (*p* = 0.045) characteristics were associated with being included in the child development, measured by the BSID-III, analysis. Being enrolled in school (*p* = 0.019) was associated with inclusion in the HOME data analysis. Finally, the following characteristics were associated with inclusion in the anthropometric growth analysis: mother enrolled in school (*p* = 0.016 for BMI-for-age, *p* = 0.008 for length-for-age), maternal history of emotional abuse (*p* = 0.016 for BMI-for-age, *p* = 0.028 for length-for-age).

## Discussion

Our study showed positive effects of a nurse HVP, the *Primeiros Laços* program, for impoverished adolescent mothers living in adverse conditions. The intervention group showed statistically significant benefits on child expressive language, as well as on two domains representing the quality of the home environment: maternal emotional/verbal responsibility and opportunities for variety in daily stimulation. To our knowledge, this is the first study testing an HVP in Brazil using an RCT design.

The impact of the intervention on child expressive language development replicates findings from one of the studies that tested the efficacy of NFP^90^. In this study, children of mothers who received visits from nurses were less likely to exhibit language delays at 21 months and had a higher development score measured by the BSID at 24 months compared with children in the control group^90^. Given that the NFP and the *Primeiros Laços* program share several components, it is not surprising that part of our results is in line with NFP findings^90^.

The observed effects of the intervention on child expressive language development could be explained by the impact of the intervention on the variety of daily stimulation and maternal emotional/verbal responsivity. More variety in daily stimulation increases the chances of expanding the child's general behavior repertoire. Moreover, it is well-known that, aside from influences of developmental changes in perception, cognition, and phonology, the development of children’s communication and language skills are supported by the way the caregivers respond to the child’s interactions^90–93^. In addition to that, longitudinal studies have demonstrated an association between variations in child language development and maternal responsiveness^93^.

The effect of *Primeiros Laços* on opportunities for variety in daily stimulation and maternal emotional/verbal responsivity may be explained by the improved parenting skills component of the intervention. Nurse home visits after children were born emphasized child development and early cognitive and socioemotional stimulation. Nurses taught participants the importance of early stimulation, specifically showing activities that can help to enhance the mother–child bond and to provide adequate stimulation for the child’s age. In line with these findings and intervention components, a previous study analyzing the present sample showed the *Primeiros Laços* program increased the probability of early stimulation behaviors by mothers at 18 months of the child’s age^94^.

Even though we found the *Primeiros Laços* program had a statistically significant effect on child language development and home environment outcomes, these effects were small to moderate according to reported Cohen’s d (0.37–0.55). This may be attributed to the fact that the *Primeiros Laços* program has intervention components divided into five domains (health and social care, environmental health, life course, parenting skills, family, and social support), not all exclusively related to child development and/or maternal caregiving skills. Moreover, participants were adolescent mothers living in adverse conditions, meaning that some problems related to food insecurity, housing, violence, among others, are prioritized in home visits, leaving less time to be dedicated to parenting skills in some cases. This may explain the small to moderate effect size on child expressive language development. Also, maternal responsivity is a complex and nuanced construct that may require more attention, time, and specific strategies to have a more significant impact on child language.

No effect of the intervention was found for oscillatory alpha power, suggesting that the home-visiting program did not result in positive effects on brain maturation by the time the children were aged 6 months. While these findings contrast with our hypotheses and previous work in children exposed to psychosocial deprivation^72,95^ the null findings are not particularly surprising given that we could not collect EEG data from the entire sample or at all of the age time-points, as we had initially planned, due to the technical difficulties with the EEG system. It may be the case that differences in oscillatory activity associated with the home-visiting program emerge later in development than age 6 months.

We did not find an effect of the *Primeiros Laços* program on child anthropometric development. This finding could be explained by political changes that influenced health indicators in the last decades. The inception of the Unified Health System *(Sistema Único de Saúde*) in 1988, a public health system with universal coverage and a focus on primary health care programs^68^ led to the drastic decline in the prevalence of stunting^96^ and low weight-for-age^97^. Data suggests that virtually every Brazilian pregnant woman have access to prenatal care (97.8%)^98^. Considering this context, it may be difficult to implement interventions that can add to the strong effects of such programs.

Our findings should be viewed in light of some limitations. First, our sample size is small, which precluded analyses to understand the effects of the intervention in specific subgroups (for example, a comparison of intervention effects in participants with higher versus lower exposure to food insecurity, housing, violence, etc.). Moreover, the small sample size precluded a mediation analysis to better understand whether there is a connection between increased maternal emotional/verbal responsivity and opportunities for variety in daily stimulation, and the expressive language improvements observed in children from the intervention group. Second, our study was powered based on the potential effect of the intervention in EEG alpha wave frequency, so our findings should be interpreted with caution, since there is a possibility that we did not find an effect of the intervention in some of the outcomes because of our sample size. Third, our assessment of the home environment, stimulation, and mother–child relationship was based exclusively on the frequency of behaviors measured by the HOME scale. We did not assess the quality of the interaction between participants and their children using a more fine-grained measure. For instance, maternal responsiveness is composed of only 11 items with binary response options (yes/no), therefore lacking a full range of maternal behaviors and dimensional detail. Likewise, we did not assess the involvement of the child's father or other father figures in the home^99^. Fourth, by the time children completed 24-months of age, 40% of the intervention group were not receiving home visits anymore and only 67.5% received at least 50% of planned home visits. The dropout rate and limited number of visits delivered can potentially explain the lack of findings on some outcomes, or the small to moderate effects found on developmental and environmental outcomes. Fifth, the randomization and allocation procedures were not conducted by an external researcher, potentially contributing to a bias in this process. However, we did not find any evidence of balancement issues favoring the intervention. Sixth, we were not able to collect oscillatory alpha power data according to our original plan due to EEG technical difficulties beyond our control. This issue significantly limited the available data for our EEG analysis, limiting our statistical power and biasing our results. Finally, baseline characteristics were associated with participants being included in our outcome analysis (e.g., maternal age, mother enrolled in school, maternal childhood trauma). This may have biased our overall findings. These characteristics should be considered in future studies similar to ours. Targeting these characteristics via intervention mechanisms could help prevent loss to follow-up.

Lastly, the long-term outcomes of our intervention remain to be determined. Future assessments of the study population will allow us to investigate whether the effects on child language development are maintained. If indeed they are, they will add to the extant literature showing the potential of HVP programs to prevent and promote child health, as rates of language impairments in the first years of life are estimated to be around 65% in children with low socio-economic background^100^. The possibility of preventing such impairments in these populations represents an enormous gain for the community, as language delay is associated with impairments in reading ability^101^, school readiness^102^, and persistent communication problems, which have been shown to negatively affect children’s development of prosocial behaviors (i.e., behaviors involving empathetic actions, as well as putting the needs of others first, and having a positive sense of social responsibility, and which are very important for children to build better social relationships when entering formal school)^102–105^. Follow-up studies will also be important to observe whether differences between intervention and control groups appear in other cognitive domains. In NFP, superior executive functioning and behavioral adaptation during testing was observed in a follow-up conducted two years after the program ended (at age 4) (Olds et al., 2004). Finally, from a public health perspective, it will be important to document in future long-term outcome studies whether or not the mothers who participated in *Primeiros Laços* went on to give birth to fewer children and if there was greater spacing between first and second pregnancies for those mothers who did have additional children^106,107^.

## Conclusions

Our findings demonstrate the efficacy of *Primeiros Laços*, a nurse HVP for pregnant adolescents living in an urban deprived area, on child expressive language, as well as on two domains representing the quality of the home environment: maternal emotional/verbal responsibility and opportunities for variety in daily stimulation. To our knowledge, our study is the first to use a randomized controlled trial design to test the efficacy of an HVP intervention on child and home environment outcomes in Brazil. *Primeiros Laços* is a promising intervention able to promote the development of children born of low-income adolescent mothers. Due to its structured and manualized nature, *Primeiros Laços* could be implemented in the context of the public health system, therefore working alongside well-established programs. Since adolescent pregnancy is a prevalent public health issue in Brazil and other LMICs, an evidence-based intervention such as *Primeiros Laços* is timely. However, considering the limitations of our study (e.g., small sample size, dropout rate) future studies should be conducted to assess *Primeiros Laços* potential to benefit adolescent mothers and their children.

## Supplementary Information

Supplementary Information.

## Abbreviations

HVP: Home visiting program
LMIC: Low- and middle-income countries
BDI: Beck Depression Inventory
BAI: Beck Anxiety Scale
ADHD: Adult Attention Deficit Hyperactivity Disorder
ASRS: ADHD Self-report Scale
CTQ: Childhood trauma questionnaire
NFP: Nurse-Family Partnership
BSID-III: Bayley Scales of Infant Development Third Edition
HOME: Home Observation for Measurement of the Environment
EEG: Electroencephalogram
GEE: Generalized estimating equation
