# Supplementary material for: A randomized controlled trial testing the efficacy of a Nurse Home Visiting Program for Pregnant Adolescents
Source: Sci Rep. 2021 Jul 13;11:14432. doi: 10.1038/s41598-021-93938-7 (PMC8277870; doi:10.1038/s41598-021-93938-7)
Supplement: Supplementary file 1 — Supplementary Information. [file 41598_2021_93938_MOESM1_ESM.docx]

**Title**: A randomized controlled trial testing the efficacy of a Nurse Home Visiting Program for Pregnant Adolescents

**Authors**: Daniel Fatori* (1), Pedro Fonseca Zuccolo (1), Elizabeth Shephard (1), Helena Brentani (1), Alicia Matijasevich (2), Alexandre Archanjo Ferraro (3), Lislaine Aparecida Fracolli (4), Anna Maria Chiesa (4), James Leckman (5), Euripedes Constantino Miguel Filho (1), Guilherme V. Polanczyk* (1)

**Affiliation**: (1) Departamento de Psiquiatria, Faculdade de Medicina FMUSP, Universidade de Sao Paulo, São Paulo, SP, BR.; (2) Departamento de Medicina Preventiva, Faculdade de Medicina FMUSP, Universidade de São Paulo, São Paulo, Brasil; (3) Departamento de Pediatria, Faculdade de Medicina FMUSP, Universidade de São Paulo, São Paulo, SP, BR; (4) Departamento de Enfermagem em Saúde Coletiva da Escola de Enfermagem, Universidade de São Paulo, São Paulo, Brasil; (5) Yale Child Study Center, Yale University School of Medicine, New Haven, USA

**Supplementary Files**

**Supplementary Methods**

Electroencephalography (EEG) Methods and Procedures

We assessed oscillatory power in the mid-range infant alpha frequency (6-9Hz) via electroencephalography (EEG) during a quasi-resting-state condition when the children were aged 6 months. This measure was selected based on previous research showing that infants exposed to psychosocial adversity (i.e. being raised in an orphanage) showed significantly reduced oscillatory alpha power at age 6-30 months compared to infants who were adopted into foster care families[^1^](https://paperpile.com/c/RBvDU9/SiQXD). Reduced alpha power in the institutionalized infants was associated with reduced grey matter volume later in development and was therefore interpreted as reflecting reduced cortical maturation as a consequence of psychosocial deprivation[^2^](https://paperpile.com/c/RBvDU9/hu7Sn). Based on these findings, we predicted that children of adolescent mothers who did not receive the intervention would show significantly reduced alpha power at age 6, 12 and 24 months compared to children of mothers who received the intervention. However, due to technical problems with the EEG system in our laboratory (hardware malfunction beyond repair, even though multiple attempts were made to repair it), it was only possible to collect EEG data from a proportion of the participants at age 6 months and from none of the participants at the later (12 and 24 month) time-points. We previously published an analysis of the 6-month EEG data in which we focused on associations with maternal psychopathology; in that paper we also reported a lack of group differences in oscillatory alpha power at age 6 months[^3^](https://paperpile.com/c/RBvDU9/JFEvs), but the analyses were conducted using a different statistical method to the analysis approach in the original trial protocol. Thus, in the current paper we report the results of the planned statistical analysis approach comparing the intervention and control children on alpha power.

EEG data were collected while children watched a 3-minute video of abstract shapes while seated on their mother’s lap ~65 cm in front of a computer screen in a dimly lit room. The data were recorded using a 128-channel Geodesic Sensor Net and a NetAmp 200 DC-coupled amplifier (Electrical Geodesics Inc., Eugene, OR). The data were referenced online to electrode Cz, sampled at 500 Hz, and bandpass filtered between 0.1 and 100 Hz. EEG data were processed offline using Brain Vision Analyzer version 2.1 (Brain Products, Munich, Germany). Electrodes around the rim of the net were contaminated by excessive artifacts and removed from all participants, leaving 80 electrodes for further processing and analysis. The data were filtered using 0.1-Hz high-pass, 60-Hz low-pass 24-dB/oct Butterworth filters with a 60-Hz notch filter for residual electrical line noise. Periods of data with excessive noise or during which the children were crying or reacting to the examiner or other external stimuli were excluded. Remaining flat or noisy channels were removed and interpolated using spherical spline interpolation prior to re-referencing to the average reference. Independent components analysis was used to identify and remove ocular artifact components. The data were segmented into 2-second non-overlapping epochs. Epochs with remaining artifacts (defined as those with amplitudes +/- 150 μv) were excluded. Children with fewer than 20 artifact-free epochs were excluded from analysis (described below). Clean epochs were transformed to the frequency domain using Fast Fourier Transform (FFT) with a 10% Hanning window to obtain estimates for absolute power in the 1-50Hz range in 1Hz frequency steps at each electrode across epochs. Absolute power in the alpha band (6-9Hz) was then computed by averaging the power estimates across frequency steps in the 6-9Hz frequency range at each electrode. Subsequently, following previous research[^1^](https://paperpile.com/c/RBvDU9/SiQXD), absolute alpha power values were averaged across clusters of electrodes in the frontal (Fz and surrounding five electrodes), central (Cz and surrounding five electrodes), right-hemisphere posterior (P8 and surrounding five electrodes), left-hemisphere posterior (P7 and surrounding five electrodes) and occipital (Oz, O1, O2) scalp regions. Alpha power in each of these clusters was used in analysis.

In total, EEG data collection was attempted from 50 children at age 6 months. Of these, 19 were excluded due to technical problems with the EEG system that resulted in corrupted data files (n=4), poor-quality recordings (n=10) or fewer than 20 artefact-free epochs (n=5). Final analyses were therefore conducted on 31 participants (17 intervention, 14 control). The groups did not differ in the number of artefact-free epochs included in analysis (intervention mean=48.41, SD=19.29; control mean=44.86, SD=19.01; t(29)=-0.51, p=0.610).

**References**

1. [McLaughlin, K. A. *et al.* Delayed maturation in brain electrical activity partially explains the association between early environmental deprivation and symptoms of attention-deficit/hyperactivity disorder. *Biol. Psychiatry* **68**, 329–336 (2010).](http://paperpile.com/b/RBvDU9/SiQXD)

2. [Sheridan, M. A., Fox, N. A., Zeanah, C. H., McLaughlin, K. A. & Nelson, C. A., 3rd. Variation in neural development as a result of exposure to institutionalization early in childhood. *Proc. Natl. Acad. Sci. U. S. A.* **109**, 12927–12932 (2012).](http://paperpile.com/b/RBvDU9/hu7Sn)

3. [Shephard, E. *et al.* Effects of maternal psychopathology and education level on neurocognitive development in infants of adolescent mothers living in poverty in Brazil. *Biological Psychiatry: Cognitive Neuroscience and Neuroimaging* (2019) doi:](http://paperpile.com/b/RBvDU9/JFEvs)[10.1016/j.bpsc.2019.05.009](http://dx.doi.org/10.1016/j.bpsc.2019.05.009)[.](http://paperpile.com/b/RBvDU9/JFEvs)

| **Table S1.** Number of participants included in efficacy analyses per group. | | |
| --- | --- | --- |
| **Outcomes** | **Group** | **Included** |
| **EEG** | **C** | 14 |
|  | **I** | 17 |
| **HOME** | **C** | 32 |
|  | **I** | 30 |
| **BSID** | **C** | 36 |
|  | **I** | 35 |
| **Body Mass Index (z-score)** | **C** | 39 |
|  | **I** | 36 |
| **Length-for-age (z-score)** | **C** | 37 |
|  | **I** | 35 |

Abbreviations: C=control group, I=intervention group.

| **Table S2**. Differences at baseline between participants included vs. not included in the efficacy analyses. Data depicted as mean (SD) or N (%). | | | | | | | | | | | | | | | |
| --- | --- | --- | --- | --- | --- | --- | --- | --- | --- | --- | --- | --- | --- | --- | --- |
|  | **BSID outcome** | | | **HOME outcome** | | | **Body Mass Index (z score) outcome** | | | **Length-for-age (z score) outcome** | | | **Electroencephalography** | | |
| **Characteristics at baseline** | **Not included** | **Included** | **p value** | **Not included** | **Included** | **p value** | **Not included** | **Included** | **p value** | **Not included** | **Included** | **p value** | **Not included** | **Included** | **p value** |
| **Maternal age** | 17.4 (0.5) | 17.1 (1.3) | 0.402 | 17.3 (0.6) | 17.1 (1.3) | 0.651 | 17.6 (0.5) | 17.1 (1.3) | 0.313 | 17.5 (0.5) | 17.1 (1.3) | 0.359 | 17.4 (0.9) | 16.6 (1.5) | **0.004** |
| **Maternal ethnicity, White** | 3 (33.3%) | 22 (31.0%) | 1.000 | 3 (27.3%) | 22 (31.9%) | 1.000 | 2 (28.6%) | 23 (31.5%) | 1.000 | 3 (37.5%) | 22 (30.6%) | 0.700 | 17 (34.7%) | 8 (25.8%) | 0.465 |
| **Mother is enrolled in school** | 0 | 35 (49.3%) | **0.004** | 1 (9.1%) | 34 (49.3%) | **0.019** | 0 | 35 (47.9%) | **0.016** | 0 | 35 (48.6%) | **0.008** | 15 (30.6%) | 20 (64.5%) | **0.005** |
| **Grandmother educational level, Illiterate/Inc. primary education** | 3 (33.3%) | 34 (47.9%) | 0.494 | 3 (27.3%) | 34 (49.3%) | 0.208 | 3 (42.9%) | 34 (46.6%) | 1.000 | 3 (37.5%) | 34 (47.2%) | 0.719 | 21 (42.9%) | 16 (51.5%) | 0.495 |
| **Maternal educational level, Illiterate/Inc. primary education** | 3 (33.3%) | 9 (12.7%) | 0.129 | 3 (27.3%) | 9 (13.0%) | 0.357 | 3 (42.9%) | 9 (12.3%) | 0.065 | 3 (37.5%) | 9 (12.5%) | 0.094 | 43 (87.8%) | 25 (80.6%) | 0.522 |
| **Maternal occupation, Working for pay** | 2 (22.2%) | 13 (18.3%) | 0.674 | 2 (18.2%) | 13 (18.8%) | 1.000 | 1 (14.3%) | 14 (19.2%) | 1.000 | 2 (25.0%) | 13 (18.1%) | 0.640 | 10 (20.4%) | 5 (16.1%) | 0.772 |
| **Family enrolled in social welfare program** | 2 (22.2%) | 19 (26.8%) | 1.000 | 3 (27.3%) | 18 (26.1%) | 1.000 | 2 (28.6%) | 19 (26.0%) | 1.000 | 2 (25.0%) | 19 (26.4%) | 1.000 | 13 (26.5%) | 8 (25.8%) | 1.000 |
| **Number of people living in the residence** | 2.8 (1.1) | 3.7 (1.7) | 0.112 | 2.9 (1.2) | 3.7 (1.7) | 0.134 | 2.8 (1.2) | 3.7 (1.7) | 0.213 | 2.7 (1.8) | 3.7 (1.7) | 0.125 | 3.5 (1.5) | 3.7 (1.7) | 0.682 |
| **Family income, 0-800 reais** | 2 (28.6%) | 13 (19.7%) | 0.627 | 2 (22.2%) | 13 (20.3%) | 1.000 | 2 (33.3%) | 13 (19.4%) | 0.596 | 2 (28.6%) | 13 (19.7%) | 0.627 | 8 (18.2%) | 7 (24.1%) | 0.566 |
| **Maternal lifetime police problems** | 2 (22.2%) | 6 (8.6%) | 0.225 | 2 (18.2%) | 6 (8.8%) | 0.308 | 1 (14.3%) | 7 (9.7%) | 0.541 | 0.1 (0.3) | 0.1 (0.3) | 0.817 | 4 (8.3%) | 4 (12.9%) | 0.705 |
| **Presence of family food insecurity** | 5 (55.6%) | 29 (40.8%) | 0.484 | 5 (45.4%) | 29 (42.0%) | 1.000 | 4 (57.1%) | 30 (41.1%) | 0.451 | 4 (50.0%) | 30 (41.7%) | 0.717 | 17 (34.7%) | 17 (54.8%) | 0.105 |
| **Maternal history of substance use** | 3 (33.3%) | 28 (39.4%) | 1.000 | 3 (27.3%) | 28 (40.6%) | 0.515 | 3 (42.9%) | 28 (38.4%) | 1.000 | 3 (37.5%) | 28 (38.9%) | 1.000 | 15 (30.6%) | 16 (51.6%) | 0.098 |
| **Presence of maternal chronic disease** | 2 (22.2%) | 6 (8.4%) | 0.220 | 2 (18.2%) | 6 (8.7%) | 0.302 | 2 (28.6%) | 6 (8.2%) | 0.143 | 2 (25.0%) | 6 (8.3%) | 0.181 | 4 (8.2%) | 4 (12.9%) | 0.704 |
| **Presence of maternal sexually transmitted disease** | 2 (22.2%) | 6 (8.4%) | 0.220 | 2 (18.2%) | 6 (8.7%) | 0.302 | 1 (14.3%) | 7 (9.6%) | 0.536 | 2 (25.0%) | 6 (8.3%) | 0.181 | 3 (6.1%) | 5 (16.1%) | 0.250 |
| **Maternal Self-efficacy** | 30.5 (2.2) | 29.4 (6.8) | 0.643 | 31.1 (6.0) | 29.3 (6.9) | 0.424 | 29.6 (7.0) | 29.6 (6.8) | 0.997 | 29.7 (6.4) | 29.5 (6.8) | 0.935 | 29.3 (7.0) | 30.0 (6.5) | 0.625 |
| **Maternal Depression ^a^** | 1 (11.1%) | 16 (22.5%) | 0.676 | 1 (9.1%) | 16 (23.2%) | 0.441 | 1 (14.3%) | 16 (21.9%) | 1.000 | 1 (12.5%) | 16 (22.2%) | 1.000 | 9 (18.4%) | 8 (25.8%) | 0.114 |
| **Maternal Suicidal Ideation** | 0 | 3 (4.2%) | 1.000 | 0 | 3 (4.3%) | 1.000 | 0 | 3 (4.1%) | 1.000 | 0 | 3 (4.2%) | 1.000 | 2 (4.1%) | 1 (3.2%) | 0.108 |
| **Maternal Anxiety ^b^** | 0 | 10 (14.1%) | 0.593 | 0 | 10 (14.5%) | 0.342 | 0 | 10 (13.7%) | 0.587 | 0 | 10 (13.9%) | 0.586 | 4 (8.2%) | 6 (19.3%) | 0.174 |
| **Maternal ADHD ^c^** | 2 (22.2%) | 9 (12.7%) | 0.603 | 2 (18.2%) | 9 (13.0%) | 0.643 | 2 (28.6%) | 9 (12.3%) | 0.245 | 2 (25.0%) | 9 (12.5%) | 0.302 | 7 (14.3%) | 4 (12.9%) | 1.000 |
| **Maternal history of emotional abuse ^d^** | 4 (44.4%) | 10 (14.1%) | **0.045** | 4 (36.4%) | 10 (14.5%) | 0.095 | 4 (57.1%) | 10 (13.7%) | **0.016** | 4 (50.0%) | 10 (13.9%) | **0.028** | 8 (16.3%) | 6 (19.3%) | 0.769 |
| **Maternal history of physical abuse ^e^** | 1 (11.1%) | 6 (8.4%) | 0.581 | 1 (9.1%) | 6 (8.7%) | 1.000 | 1 (14.3%) | 6 (8.2%) | 0.487 | 1 (12.5%) | 6 (8.3%) | 0.536 | 1 (2.0%) | 6 (19.3%) | **0.012** |
| **Maternal history of sexual abuse ^f^** | 0 | 5 (7.0%) | 1.000 | 0 | 5 (7.2%) | 1.000 | 0 | 5 (6.8%) | 1.000 | 0 | 5 (6.9%) | 1.000 | 3 (6.1%) | 2 (6.4%) | 1.000 |
| **Maternal history of emotional neglect ^g^** | 1 (11.1%) | 8 (11.3%) | 1.000 | 1 (9.1%) | 8 (11.6%) | 1.000 | 1 (14.3%) | 8 (11.0%) | 0.581 | 1 (12.5%) | 8 (11.1%) | 1.000 | 5 (10.2%) | 4 (12.9%) | 0.729 |
| **Maternal history of physical neglect ^h^** | 1 (11.1%) | 5 (7.0%) | 0.523 | 1 (9.1%) | 5 (7.2%) | 1.000 | 1 (14.3%) | 5 (6.8%) | 0.434 | 1 (12.5%) | 5 (6.9%) | 0.480 | 2 (4.1%) | 4 (12.9%) | 0.200 |
| ^a^ BDI moderate or severe levels (score >20), ^b^ BAI moderate or severe levels (score >16), ^c^ ASRS clinical level (score >9), ^d^ CTQ moderate or severe levels (score >13), ^e^ CTQ moderate or severe levels (score >10), ^f^ CTQ moderate or severe levels (score >8), ^g^ CTQ moderate or severe levels (score >15), ^h^ CTQ moderate or severe levels (score >10).  Abbreviation: ADHD=attention-deficit/hyperactivity disorder, ASRS=ADHD Self-report scale, BAI=Beck Anxiety Inventory, BDI=Beck Depression Inventory, BSID=Bayley Scales of Infant Development, CTQ=Childhood trauma questionnaire, Inc=incomplete. | | | | | | | | | | | | | | | |

| **Table S3.** Frequencies of length/height-for-age and BMI-for-age by time point. | | | | | | | | | | | | | |
| --- | --- | --- | --- | --- | --- | --- | --- | --- | --- | --- | --- | --- | --- |
| **Anthropometric outcomes** | **Categories** | **Birth** | | | **6 mo** | | | **12 mo** | | | **24 mo** | | |
|  |  | **C** | **I** | **T** | **C** | **I** | **T** | **C** | **I** | **T** | **C** | **I** | **T** |
| **Length/Heigth-for-age (z score)** | -3 to -2 | 4 (40.0%) | 6 (60.0%) | 10 (14.7%) | 2 (66.7%) | 1 (33.3%) | 3 (4.8%) | 2 (100%) | 0 | 2 (4.0%) | 3 (50.0%) | 3 (50.0%) | 6 (13.6%) |
|  | -1 to 3 | 31 (53.4%) | 27 (46.5%) | 58 (85.3%) | 31 (51.7%) | 29 (48.3%) | 60 (95.2%) | 24 (50%) | 24 (50%) | 48 (96.0%) | 20 (52.3%) | 18 (47.4%) | 38 (86.4%) |
| **BMI-for-age**  **(z score)** | -3 to -2 | 0 | 2 (100%) | 2 (2.9%) | 0 | 1 (100%) | 1 (1.6%) | 21 (53.8%) | 18 (46.1%) | 0 | 1 (100%) | 0 | 1 (2.4%) |
|  | -2 to 1 | 31 (53.4%) | 27 (46.5%) | 58 (85.3%) | 29 (58.0%) | 21 (42.0%) | 50 (82.0%) | 0 | 0 | 39 (81.2%) | 11 (45.8%) | 13 (54.2%) | 24 (57.1%) |
|  | 1 to 3 | 4 (50.0%) | 4 (50.0%) | 8 (11.8%) | 4 (40.0%) | 6 (60.0%) | 10 (16.4%) | 4 (44.4%) | 5 (55.6%) | 9 (18.7%) | 9 (52.9%) | 8 (47.1%) | 17 (40.5%) |
| Abbreviations: C=control group, I=intervention group, T=total sample. | | | | | | | | | | | | | |
